# Loneliness and Hypervigilance to Social Cues in Females: An Eye-Tracking Study

**DOI:** 10.1371/journal.pone.0125141

**Published:** 2015-04-27

**Authors:** Gerine M. A. Lodder, Ron H. J. Scholte, Ivar A. H. Clemens, Rutger C. M. E. Engels, Luc Goossens, Maaike Verhagen

**Affiliations:** 1 Behavioural Science Institute, Radboud University, Nijmegen, The Netherlands; 2 Donders Institute for Brain, Cognition, and Behaviour, Radboud University, Nijmegen, The Netherlands; 3 Research Group School Psychology and Child and Adolescent Development, KU Leuven, Leuven, Belgium; University of Florida, UNITED STATES

## Abstract

The goal of the present study was to examine whether lonely individuals differ from nonlonely individuals in their overt visual attention to social cues. Previous studies showed that loneliness was related to biased post-attentive processing of social cues (e.g., negative interpretation bias), but research on whether lonely and nonlonely individuals also show differences in an earlier information processing stage (gazing behavior) is very limited. A sample of 25 lonely and 25 nonlonely students took part in an eye-tracking study consisting of four tasks. We measured gazing (duration, number of fixations and first fixation) at the eyes, nose and mouth region of faces expressing emotions (Task 1), at emotion quadrants (anger, fear, happiness and neutral expression) (Task 2), at quadrants with positive and negative social and nonsocial images (Task 3), and at the facial area of actors in video clips with positive and negative content (Task 4). In general, participants tended to gaze most often and longest at areas that conveyed most social information, such as the eye region of the face (T1), and social images (T3). Participants gazed most often and longest at happy faces (T2) in still images, and more often and longer at the facial area in negative than in positive video clips (T4). No differences occurred between lonely and nonlonely participants in their gazing times and frequencies, nor at first fixations at social cues in the four different tasks. Based on this study, we found no evidence that overt visual attention to social cues differs between lonely and nonlonely individuals. This implies that biases in social information processing of lonely individuals may be limited to other phases of social information processing. Alternatively, biased overt attention to social cues may only occur under specific conditions, for specific stimuli or for specific lonely individuals.

## Introduction

Loneliness, defined as a negative emotional response to a discrepancy between the desired and actual quality or quantity of interpersonal relationships [1], can have severe consequences for physical and mental health, including higher morbidity and mortality [2]. Earlier research suggested that loneliness may be related to heightened attention for social cues in the environment, which can be used to prevent social rejection and promote opportunity for inclusion [3,4,5]. Various types of psychopathology related to loneliness such as depression and social anxiety have also been linked to biased processing of social information. Depression is mainly linked to biases in post-attentive processing such as remembering negative events more clearly, and social anxiety is primarily linked to selective visual attention to negative social cues (i.e., hypervigilance for negative social cues) [6]. So far, research on the link between loneliness and processing of social cues has predominantly focused on the post-attentive processing stage. For instance, loneliness has been linked to negative interpretation of social cues and enhanced memory for social cues [4,7]. However, research on the relation between loneliness and visual attention (i.e., gazing at social cues), which is an earlier step of social information processing, is very limited. It is thus unclear whether biased perception of social cues in lonely individuals is merely related to biased post-attentive processing of (negative) social cues, similar to depression, or also to biased visual attention to negative social cues, similar to social anxiety [8]. In order to gain insight into this stage of social information processing, the goal of the present study was to examine whether lonely individuals show hypervigilance to negative social cues in terms of increased overt visual attention to social cues.

Social information processing consists of several steps that are interrelated [9]. Roughly, a distinction can be made between pre-attentive evaluation of cues (e.g., automatic detection of threatening cues in the peripheral vision), the allocation of attention to cues (e.g., overt visual attention or gazing at cues, and covert attention to cues), post-attentive evaluation of cues (e.g., comparing information with memory and interpretation), and the response to cues (e.g., sustained attention to the cue) [8,9]. Biased processing of social information can occur at each stage of social processing. Biases in different stages of social information processing have been linked to distinct forms of psychopathology, indicating that different mechanisms of biased information processing may underlie each of these forms of psychopathology [6,8]. This distinction is important, because knowledge of the stages in which biased processing of social cues occurs may lead to intervention opportunities. For example, if loneliness is indeed related to biased visual attention to negative social information, training lonely individuals to attend to these cues less (i.e., attention modification training) [10] may be a useful tool, whereas this is not the case if loneliness is not related to biased attention to social cues.

Social needs models of loneliness indicate that the need to belong, a fundamental need to initiate and maintain close relationship with others, is at the core of loneliness, because loneliness arises when this need is not met [4,11–13]. Feelings of unsatisfactory social connections may instigate the tendency to restore the level of belongingness [14]. One way in which individuals can restore their level of belonging is by changing the way they attend to, perceive, and react to social cues in their environment [4,11]. Social cues can inform an individual about potential rejection or acceptance. According to the hypervigilance theory, lonely individuals may have a heightened focus specifically on negative social cues, because these cues signal rejection [3,11,15,16]. Research has provided ample evidence that loneliness is related to biases in post-attentive processing of social cues. For instance, loneliness was related to increased memory for both positive and negative social events [4], more negative expectations about the way one is perceived [17,18] and more negative perceptions of social interactions [19,20]. Research on one of the first steps of social processing, overt visual attention to (i.e., gazing at) social cues, is very limited [5]. It is therefore unclear whether loneliness is exclusively related to enhanced memory for and negative interpretation of social cues, or also to hypervigilance to negative social information (i.e., biased visual attention to negative social cues).

On the one hand, based on the hypervigilance theory for loneliness, we could assume that loneliness is indeed related to hypervigilance for negative social cues [3,11]. Biases in post-attentive cognitive processing of negative social information that are apparent in lonely individuals may in fact be the result of increased visual attention to these negative cues. Earlier research showed that people show increased processing of visual stimuli that are gazed at, compared to stimuli that are not gazed at [21]. Thus, lonely individuals might give more overt visual attention to negative social cues, which elicits increased processing which may in turn explain negative interpretation of and increased memory for these cues by lonely individuals [4]. In line with this, a study examining gazing behavior at playground video scenes showed that higher loneliness was related to a higher likelihood to fixate first on rejection cues rather than other cues, which held especially for the loneliest individuals [5]. Children who were extremely lonely were found to have difficulties disengaging from rejection cues [7]. This suggests that loneliness may be related to biased overt visual attention to negative social information, in addition to biases in post-attentive processing.

On the other hand, biased processing of social cues in post-attentive processing by lonely individuals may not be the result of increased overt visual attention to social cues, but rather originate in later steps of information processing. Although the location the eyes are directed at is certainly related to what is given conscious attention to [22], shifts in attention are not necessarily accompanied by shifts in gaze [23]. Earlier research showed that differences in overt visual attention could not explain enhanced memory for emotional cues compared to neutral cues [24]. In addition, threatening cues in the environment seem to be processed by the amygdala independent of whether overt visual attention was given to these cues or not [21]. This indicates that biased post-attentive processing of certain cues is not necessarily the result of biased visual attention to these cues. Indeed, interpretation of social cues is influenced not only by the amount of overt visual attention that was given to these cues, but also by expectancies and attitudes of the perceiver [9]. Although little is known about biases in visual attention to social cues by lonely individuals, earlier studies showed that for instance in depression and borderline personality disorder, biases only occur in later stages of social information processing [6,25]. The same could be true for lonely individuals. Thus, biased processing of social cues in lonely individuals may not be due to hypervigilance for these cues, but rather originate in post-attentive processing.

### The present study

In the present study, we examined whether lonely and nonlonely individuals differ in their hypervigilance to negative social cues. We used eye-tracking equipment to measure participants' gazing behavior at social cues. Eye-tracking is highly suitable to objectively assess the ways in which individuals look at—rather than interpret and represent—social cues [26]. We mainly focused on overt visual attention to facial expressions. Emotions, or more specifically facial expressions, reflect the state of mind of the person expressing them, and may thus provide information about opportunities for inclusion (for emotions with a positive valence) or provide a warning for rejection (for emotions with a negative valence) [27,28]. The face conveys these emotions and is therefore considered to be the most important source of social information [29,30].

We used four tasks to examine differences in overt visual attention to social cues between lonely and non-lonely participants. In the first task, the Face Task, we presented participants with images of neutral faces and emotional faces. Non-clinical samples of adults tend to spend most time gazing at the eyes, nose, and mouth region of the face, of which the eyes seem to be the most important to convey social information [30–32]. As of yet, it is unknown if lonely individuals focus on different aspects of faces than nonlonely individuals. We examined whether gazing at the eye, nose and mouth region differed between lonely and non-lonely participants. Increased attention to emotion rich areas of the face, such as the eyeregion, could be a sign of hypervigilance for social cues [33].

In the second task, the Emotion Array Task, we simultaneously showed participants an array of four faces, expressing anger, fear, happiness, and a neutral expression. We examined whether participants differed in their overt visual attention to each of these emotions and the neutral expression. Increased visual attention to angry and fearful faces, which are considered to be threatening social cues [30], could be an indication of hypervigilance for threatening social information. In the third task, the Social and Nonsocial Array Task, we used an array of four different types of images, namely positive-social images, negative-social images, positive-nonsocial images, and negative-nonsocial images. Earlier research using comparable stimuli showed that the visual cortex was more strongly activated in lonely than in nonlonely people when viewing negative social images [3]. We extended this research by examining whether lonely individuals show increased overt visual attention to these cues as well, which would be a sign of hypervigilance for negative social information.

In the fourth and final task, the Video Task, we showed participants a range of video clips with a positive or negative valance, involving interactions between people. We examined whether loneliness was related to the degree to which participants gazed at the facial area of the actors in the video clips, which would be a sign of hypervigilance to social cues. Additionally, we examined whether gazing behavior differed between videos with positive or negative content.

Because depression and social anxiety are highly correlated with loneliness, and are related to biased processing of social information, we controlled for these constructs in all analyses [6,34]. We had no theoretical reason to assume that the relationship between loneliness and overt visual attention to social cues may differ between males and females [11]. In addition, most studies find no relation between loneliness and gender [35]. To increase homogeneity in the sample, we therefore used a sample consisting of females only.

## Methods

### Participants

Participants were recruited from a pool of college students who completed an online questionnaire that was designed as a selection questionnaire for multiple studies. The questionnaire was filled out by 515 students in exchange for course credit. The eye-tracking study took place approximately 2 months after completion of the prescreen questionnaire. In total, 25 nonlonely participants (scoring within the 13% lowest scores within our sample) and 26 lonely participants (scoring within the 10% highest scores within our sample) agreed to participate. The sample comprised female students from social sciences from the Radboud University Nijmegen (The Netherlands) with normal or corrected-to normal eye vision mainly. Calibration could not be completed for one lonely participant, therefore, the final sample consisted of 25 lonely and 25 nonlonely participants. Due to problems in calibration and time-constraints, one lonely and one nonlonely participant were unable to take part in the fourth task (Video Task). Age ranged from 18 to 24 years (*M* = 19.88, *SD* = 1.41). Lonely and nonlonely participants did not differ in age.

### Procedure

We invited participants into a laboratory at the research institute, where participants were seated in front of a computer screen. Four eye-tracking tasks were completed by the participants. Before each task, we ran a 13-point calibration procedure, with the calibration points being presented in random order. The four tasks were always presented in the same order. Stimuli within tasks were presented in a random order, which was determined using the Random Number Generator in SPSS. Due to time constraints, Task 4 (Video Task) was not completed by one lonely and one nonlonely participant. After calibration, we instructed the participants to “look at the images comfortably, as if you were watching TV”. The four eye-tracking tasks were separated by distraction tasks in which we asked participants to choose between different types of candy and interior designs. In addition, participants were allowed to walk around the room after each task was completed. After the eye-tracking tasks were completed, which took approximately 30 minutes in total, participants completed a questionnaire.

We obtained written informed consent from all participants involved in the study. The study was approved by the Radboud University’s IRB (Ethics Committee Social Sciences).

#### Task 1 (Face Task)

In the first task, participants viewed 50 images from the Radboud Faces Database [36] in order to examine what area of the face participants gave most visual attention to. These images portrayed 10 individuals (5 males and 5 females), each displaying 4 basic emotions (i.e., happiness, fear, anger, and sadness) and a neutral face. We used 30 additional photos from the same actors (displaying disgust, contempt and surprise) as filler items [37]. All models in the photos were Caucasian, wore a black shirt, and had their hair pulled back. The images were shown for 5 seconds, preceded by a fixation cross that was shown for 1 second. We instructed participants to move their eyes to this fixation cross whenever it appeared.

#### Task 2 (Emotion Array Task)

In the second task, we compared overt visual attention to several emotions. Participants viewed 22 arrays of 4 images from the Radboud Faces Database [36]. Each array consisted of 4 images from the same actor displaying 3 motions (anger, fear, and happiness) and a neutral face. We selected different actors than the actors used in Task 1 (Face Task), (11 male, 11 female). The arrays were presented for 8 seconds, and were preceded by a fixation cross for 1 second.

#### Task 3 (Social and Nonsocial Array Task)

In the third task, we examined preference for positive and negative, social and nonsocial images. Participants viewed 20 arrays of 4 images from the International Affective Pictures System (IAPS) [38]. All arrays contained a positive social image (e.g., playing children), a negative social image (e.g., a robbery), a positive nonsocial image (e.g., a cake) and a negative nonsocial image (e.g., a dirty bucket). Images were considered social if they contained at least 2 living humans, and were not sexually arousing in nature. Images were considered nonsocial if they contained no humans, and no animals in social interaction (e.g., a polar bear with a cub). We selected images based on valence and arousal measures of a previous study [38]. Images were included if they were within 1.5 SD of the mean of arousal ratings by females, and if they were in the top 33.3% (positive) and bottom 33.3% (negative) of valence ratings by females (mean arousal +/- 1.5 sd) see [38]. Slide numbers for the included images are included as S1 Table Arrays were shown for 10 seconds separated by a fixation cross for 1 second.

#### Task 4 (Video Task)

In the fourth task, we examined overt visual attention to social cues in dynamic images. Participants viewed 10 positive and 10 negative fragments of English-language television shows that were never broadcasted or not broadcasted at the time in the Netherlands (e.g., Make it or break it). Each fragment lasted between 29.71 and 32.61 seconds (*M* = 30.36; *SD* = .62) and consisted of 890 to 977 frames (*M* = 907.05; *SD* = 18.68). The video clips showed two or more actors that were having a conversation with positive or negative content and included sound. The fragments were selected from 53 scenes (24 positive and 29 negative) that were independently rated by three observers on valence (content, tone of voice, and facial expressions), and the absence of distracting elements (e.g., cleavage or opening credits). The most positive and negative fragments without distracting elements were selected for the study.

### Apparatus

Participants’ heads were secured so that they would not move during the eye-tracking procedure, and their eye position was fixed at 50 cm from the computer screen. Stimuli were shown on a computer screen with a resolution of 1024 x 768 pixels. Participants’ eye-movements were recorded using an Iview X Hi-Speed 500/1250 eye tracker (SMI, Teltow, Germany). Measures were taken at a 500 Hz rate, by measuring the position of the pupil relative to the corneal reflection.

Eye position samples in which the eyes exceeded a 45°/s velocity threshold were marked as saccades [39]. In order to include the on- and offset of a saccade, we also marked samples where eye velocity was increasing (i.e., acceleration was positive) before and after every marked region. Stable gaze intervals between the saccades that lasted longer than 100 ms defined a fixation and thus served as input for the analyses of visual attention. Saccades were only detected in order to identify fixations, and were not analyzed further. Furthermore, we did not analyze the first 150 ms following each new stimulus, or camera switch (Task 4—Video Task). Earlier research showed that it takes approximately 150 ms to shift attention from one spatial location to another [40]. Because participants’ eyes were still in the location of the fixation cross when a new stimulus appeared, or in the location of a previous shot following a camera switch, we did not analyze these first 150 ms.

### Measures

#### Loneliness

Loneliness was measured during the selection procedure and after the eye-tracking tasks using a Dutch translation of the UCLA Loneliness Scale Version 3 [41], which consists of 20 items that measure feelings of loneliness and connectedness (e.g., “How often do you feel left out?”). Participants rated every item on a 4-point scale (1 = *never* to 4 = *always*), with higher scores reflecting higher feelings of loneliness. Nine items were reverse coded. Loneliness was measured both in the selection questionnaire, and after the eye-tracking tasks. Reliability was high (α = .90) and after the eye-tracking tasks (α = .94).

#### Depression

To measure depressive symptoms, a Dutch translation of the Center for Epidemiologic Studies-Depression scale (CES-D) was used [42]. The CES-D consists of 20 items that measure the frequency of depressive symptoms during the past week (e.g., “In the last week I felt that everything I did was an effort”). The items were measured on a 5-point scale ranging from 0 (*rarely or none of the times = less than 1 day)* to 4 (*most or all of the time = 5–7 days*), with higher scores reflecting more feelings of depression. Four items were reverse coded (α = .90). One item in the CES-D describes feelings of loneliness (i.e., “I felt lonely”). Because overlap of the scale with and without this item was extremely high (*r* = .999, *p* = <.001) we decided to run analysis with the original CES-D scale.

#### Social anxiety

Social anxiety was measured using the Social Phobia Inventory (SPIN) [43]. The SPIN consists of 17 items that measure characteristics of social anxiety consisting of fear, avoidance, and physical reactions (e.g., “I avoid talking to people I don’t know”). Participants indicated how often they felt or behaved in a certain way during the past week on a scale ranging from 0 (*not at all*) to 4 (*extremely*), with higher scores reflecting more feelings of social anxiety (α = .92).

#### Eye-tracking measures

In Task 1 (Face Task), areas of interest were the eyeregion of the face (including eyebrows), the nose, and the mouth, because these areas convey most social information [32] In Task 2 (Emotion Array Task) and 3 (Social and Nonsocial Array Task), each image of each quadrant was an area of interest (AOI, e.g., in Task 2 (Emotion Task), the AOI “happy” was the entire image showing an actor with a happy expression). In Task 4 (Video Task), areas of interest were determined for each frame (total 18,141 frames). We used a bounding box around the facial area as an area of interest. Gazing behavior was only identified for frames in which an area of interest was visible.

We calculated two eye-tracking measures. *Total fixation duration* is the total time a participant gazed at a specific AOI stimulus in ms. *Number of fixations* is the number of times a participant fixated on a specific AOI of a single stimulus. In Task 1 (Face Task), we calculated both eye-tracking measures separately for each emotional expression, and aggregated them across trials. Eye-tracking measures were corrected for the total size of each area of interest (i.e., the eye region was larger than the nose-region, measures reported are corrected for these differences). In Tasks 2 (Emotion Array Task) and 3 (Social and Nonsocial Task), measures were aggregated across trials for each type of image (i.e., happy, fearful, angry, and neutral for Task 2, and positive-social, negative-social, positive-nonsocial, and negative-nonsocial for Task 3). We also calculated *first fixation* for these tasks, indicating the percentage of trials in which participants’ first fixation was on each of the image types. In Task 4 (Video Task), we aggregated fixations across all positive fragments and across all negative fragments.

### Analyses

We used a Repeated Measures ANOVA design to analyze all tasks. We analyzed each task separately, with stimuli (e.g., emotion) as a within subject factor and loneliness as a between subject factor. Besides descriptive statistics, results for covariates (depressive symptoms and social anxiety symptoms, both standardized) are included as (S2 Table). Results were similar when we did not include these covariates, with the one exception reported in the text. In addition, this table shows the error variances for all measures.

If Mauchly’s test of sphericity yielded a significant result, the results are reported with Greenhouse-Geisser correction for ε < .75, and with Huynh-Feldt correction for ε ≥ .75 (cf. Field, 2009). For all significant main and interaction effects, we rank ordered factors according to their means and used repeated contrasts to establish which elements differed. For instance, in Task 1 (Face Task) we rank ordered emotions by gaze duration, and contrasted the emotion that was gazed at most often to the emotion that was gazed at second most often and so forth [44].

## Results

### Descriptive Statistics

Tables 1 and 2 depict means and standard deviations for number of fixations and fixation duration for lonely and nonlonely participants in each task. T-tests showed that lonely participants had higher social anxiety scores (*t*(45) = 4.54, *p* < .001) and higher depression scores (*t*(45) = 5.07, *p* < .001) than nonlonely participants. We therefore controlled for depression and social anxiety in all further analyses by adding them as covariates to the analyses. The loneliness scores that were collected after the eye-tracking tasks correlated highly with loneliness scores measured for selection purposes (*r* = .93, *p* < .001), indicating that rank-order stability of loneliness across a 2-month period was relatively high. Loneliness measured after the eye-tracking tasks significantly differed between lonely and non-lonely individuals (*t* (34.47) = 17.44, *p* < .001). Means and *SD* for first fixations in Task 2 (Emotion Array Task) and 3 (Social and Nonsocial Array Task) are included as S3 Table).

**Table 1:** Means and Standard Deviations for Gaze Duration in ms and Number of Fixations for Total Sample, Lonely and Nonlonely Participants for Task 1.

Emotion	AOI	Gaze Duration						Number of Fixations					
		Total		Lonely		Nonlonely		Total		Lonely		Nonlonely	
		*M*	*SD*	*M*	*SD*	*M*	*SD*	*M*	*SD*	*M*	*SD*	*M*	*SD*
Anger	Eyes	18.91	7.89	18.42	9.13	19.41	6.57	17.65	7.31	17.4	8.55	17.9	5.98
	Nose	11.03	7.02	11.68	6.77	10.38	7.35	11.66	7.01	12.42	6.85	10.9	7.23
	Mouth	5.81	4.88	6.22	5.41	5.4	4.36	6.19	4.61	6.58	5.03	5.8	4.21
Fear	Eyes	15.55	6.17	15.48	7.28	15.62	4.96	14.77	5.49	14.64	6.58	14.9	4.27
	Nose	9.33	6.83	9.98	6.78	8.67	6.96	10.13	7.12	11.26	7.16	8.99	7.03
	Mouth	5.02	4.4	5.37	4.71	4.68	4.12	4.93	3.99	5.55	4.51	4.31	3.36
Happiness	Eyes	16.15	6.74	15.85	8.28	16.45	4.9	15.12	6.2	14.64	7.61	15.6	4.49
	Nose	10.4	7.86	10.14	8.23	10.66	7.63	10.99	7.3	11.27	7.93	10.71	6.76
	Mouth	6.38	4.24	6.54	4.95	6.22	3.47	6.42	3.97	6.82	4.72	6.02	3.07
Sadness	Eyes	16.83	6.69	15.72	7.86	17.94	5.21	15.76	6.33	14.7	7.33	16.81	5.08
	Nose	11.33	8.52	12.76	8.82	9.89	8.13	11.77	8.41	13.25	8.78	10.29	7.91
	Mouth	5.61	4.84	6.7	5.77	4.53	3.48	5.75	4.45	7.01	5.04	4.49	3.42
Neutral	Eyes	17.12	7.41	17.11	8.93	17.13	5.68	16.07	6.74	15.89	8.13	16.24	5.15
	Nose	10.28	8.44	10.32	8.7	10.24	8.35	10.98	8.05	12.04	8.47	9.91	7.61
	Mouth	5.89	4.76	6.92	5.62	4.86	3.52	5.85	4.38	6.99	4.98	4.71	3.41

*N* = 25 for both lonely and nonlonely group. Task 1 is the Face Task.

**Table 2:** Means and Standard Deviations for Gaze Duration in ms and Number of Fixations for Total Sample, Lonely and Nonlonely Participants for Tasks 2, 3 and 4.

Task	AOI	Gaze Duration						Number of Fixations					
		Total		Lonely		Nonlonely		Total		Lonely		Nonlonely	
		*M*	*SD*	*M*	*SD*	*M*	*SD*	*M*	*SD*	*M*	*SD*	*M*	*SD*
Task 2	Anger	1849.49	1349.19	1342.24	244.74	1356.14	173.91	6.2	4.45	4.33	0.8	4.57	0.77
	Fear	2221.55	1568.59	1544.58	306.54	1592.61	285.89	7.14	5.22	5.06	1.06	5.38	1
	Happiness	2485.76	1710.59	1722.26	343.54	1698.92	340.29	7.45	5.46	5.3	1.01	5.62	0.87
	Neutral	2085.76	1491.96	1546.78	325.22	1437.14	239.26	6.86	4.89	4.83	0.94	4.96	1.08
Task 3	Pos Soc	1325.56	3539.34	2391.81	491.99	2448.09	507.26	5.24	12.79	8.7	1.49	9.24	1.96
	Neg Soc	679.7	2503.01	2256.17	490.55	2217.49	549.15	2.75	9.06	8.31	1.46	8.46	1.82
	Pos NonSoc	1216.7	3272.47	1629.21	489.92	1433.22	411.2	5.5	11.64	5.94	1.69	5.47	1.44
	Neg NonSoc	570.83	1612.9	1150.03	274.99	1150.59	295.22	2.33	6.1	4.36	0.94	4.36	1.03
Task 4	Positive	11977.9	5894.14	10946.7	6163.15	13009.2	5548.73	33.99	15.71	29.89	16.12	38.08	14.46
	Negative	13999.4	6567	12979.8	7072.15	15018.9	5995.11	38.28	15.96	33.75	17.61	42.8	12.95

*N* = 25 for lonely and nonlonely group in Tasks 2 and 3. *N* = 24 for lonely and nonlonely group in Task 4. Task 2 = Emotion Array Task. Task 3 = Social and Nonsocial Array Task. Task 4 = Movie Task.

### Task 1 (Face Task)

**Fig 1:**
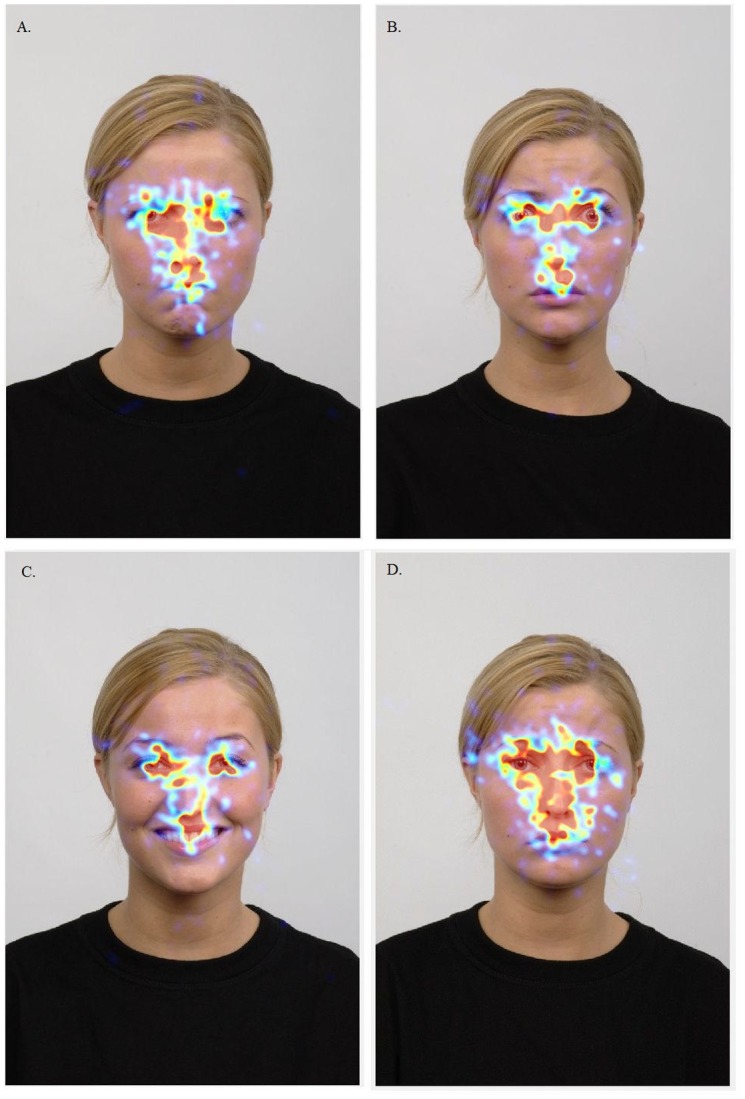
Heatmaps for gazing behavior across all participants for four emotions. Example heatmap for gazing behavior at different emotional expressions, namely (A) Angry (B) Fearful (C) Happy and (D) sad.

Table 3 shows the results of a 5 (Within Subject; Emotion; anger, fear, happy, sad, and neutral) by 3 (Within Subject; AOI; mouth, nose, eyes) by 2 (Between Subjects; lonely vs. nonlonely) Repeated Measures ANOVA. This analysis was performed to examine whether gazing behavior differed between emotions, AOIs, and between loneliness groups. Results revealed similar findings for gaze duration and number of fixations. Participants gazed longer and more often at some AOI’s than at others (eyes > nose > mouth) (see S1 Fig). Differences in gazing behavior were also found between emotions (anger > [sad = neutral = happy] > fear). The AOI by Emotion interaction showed that differences in gazing behavior between areas of interest were not equal between emotions. The difference between gazing behavior at the eyes compared to the nose were stronger in images showing angry emotions than other emotions (anger > [sad = neutral = happy = fear]). Differences in gazing behavior between nose and mouth were equal between all emotions. There were no main effects or interaction effects for loneliness groups. This indicates that, although there were differences in gazing behavior between emotions and AOIs for the entire sample, gazing behavior did not differ between lonely and nonlonely participants. Fig 1 shows an example for gazing behavior at four emotions. Results were similar when we included the filler items (i.e., contempt, disgust, and surprise) in the analyses.

**Table 3:** Repeated Measures ANOVA Results for Gaze Duration in ms and Number of Fixations for All Tasks.

Task	Effect	Fixation Duration				N Fixations			
		*df*	*MS*	*F*	η2	*df*	*MS*	*F*	η2
Task 1	Loneliness	1	0.2	0.02	0	1	2.43	0.22	0
	AOI	1.78	884.6	38.49***	0.46	1.67	7558.6	33.92***	0.42
	AOI x Lone	1.78	6.31	0.03	0	1.67	4.4	0.18	0
	Emotion	4	74.32	16.59***	0.27	4	68.35	2.37***	0.31
	Emo x Lone	4	2.99	0.67	0.01	4	3.91	1.16	0.02
	AOI x Emo	7.55	25.95	2.64**	0.05	7.63	16.57	2.27*	0.05
	AOI x Emo x Lone	7.55	14.72	1.5	0.03	7.63	9.34	1.28	0.03
Task 2	Loneliness	1	8971.87	0.3	0.01	1	0.74	1.46	0.03
	Emotion	2.82	1208253.08	16.42***	0.26	2.95	9.69	18.06***	0.28
	Emotion x Lone	2.82	43094.94	0.59	0.01	2.95	0.16	0.3	0.01
Task 3	Loneliness	1	19356.33	0.39	0.01	1	0.1	0.14	0
	Image	2.77	1922352.4	87.71***	0.66	2.75	261.12	109.88***	0.7
	Image x Lone	2.77	199939.75	0.91	0.02	2.75	2.62	1.1	0.02
Task 4	Lone	1	9371122.41	0.24	0.01	1	621.65	2.56	0.05
	Valence	1	98067836.94	86.87***	0.66	1	441.96	7.29***	0.62
	Valence x Lone	1	1613139.35	1.43	0.03	1	4.5	6.44*	0.13

All analyses were controlled for the effects of depression and social anxiety. These results are presented as supporting material, alongside error variances for all analyses (S2 Table).

* *p* < .05.

** *p* < .01.

*** *p* < .001. Emo = Emotion. Lone = loneliness. Task 2 = Emotion Array Task. Task 3 = Social and Nonsocial Array Task. Task 4 = Movie Task.

### Task 2 (Emotion Array Task)

Table 3 shows the results of a 4 (Within Subjects; anger, fear, happiness, and neutral) by 2 (Between Subjects; lonely vs. nonlonely) Repeated Measures ANOVA. Repeated contrasts showed that participants’ gaze duration was different for different emotions in the (happiness > [fear = neutral] > anger) (see S2 Fig). For the number of fixations, we also found differences between emotions ([happiness = fear] > neutral > anger). Again, there were no main effects or interaction effects for loneliness, indicating that lonely and nonlonely participants had similar gazing behavior at the different emotions in terms of both gaze duration and number of fixations. In addition, we compared the number of times participants had their first fixation on each of the emotions (see Table 4). Results showed a small but significant effect, indicating that participants were less likely to first look at happy faces than all other faces ([fear = neutral = angry] > happiness). There were no differences between groups, indicating that lonely and non-lonely participants had similar first fixations.

**Table 4:** Repeated Measures ANOVA Results for Percentage of First Fixations for Task 2 (Emotional Array Task) and 3 (Social and Nonsocial Array Task).

Task	Effect	*Df*	*MS*	*F*	η2
Task 2	Social Anxiety	1	0	0.31	0.01
	Depression	1	0	3.03	0.06
	Loneliness	1	0	4.05	0.08
	Error (between)	46	0		
	Emotion	3	21.46	4.85**	0.1
	Emotion x Soc Anx	3	1.97	0.44	0.01
	Emotion x Depression	3	1.11	0.25	0.01
	Emotion x Loneliness	3	3.21	0.73	0.02
	Error (emotion)	138	4.42		
Task 3	Social Anxiety	1	0	0	0
	Depression	1	0	0.69	0.02
	Loneliness	1	0	0	0
	Error (between)	44	0		
	Image	2.96	5994.48	49.10***	0.52
	Image x Soc Anx	2.96	131.39	1.08	0.02
	Image x Depression	2.96	50.18	0.41	0.01
	Image x Loneliness	2.96	48.79	0.4	0.01
	Error (Image)	136.23	122.08		

** *p* < .01.

*** *p* < .001.

### Task 3 (Social and Nonsocial Array Task)

Table 3 displays results for a 4 (Within Subject; Image; positive-social, positive-nonsocial, negative-social, and negative-nonsocial) by 2 (Between Subject; lonely vs. nonlonely) Repeated Measures ANOVA. Results indicate that participants showed different gazing behavior at positive, negative, social, and nonsocial images ([positive social = negative social] > positive nonsocial > negative nonsocial), for both gaze duration and number of fixations (see S3 Fig). Main effects and interaction effects for loneliness did not yield significance, indicating that lonely and nonlonely participants had similar gazing patterns at different types of images. Additionally, we looked at differences between lonely and non-lonely participants in first fixations on each of the images (see Table 4). Results showed that there was a main effect for type of image (Positive Social > Negative Social > Positive Nonsocial > Negative Nonsocial). No differences occurred between lonely and non-lonely participants. Thus, participants’ first fixations in both the lonely and nonlonely group were most often at the positive social images.

### Task 4 (Video Task)

Table 3 shows the results of a 2 (Within Subjects; positive vs. negative valence) by 2 (Between Subjects; lonely vs. nonlonely) Repeated Measures ANOVA. Results indicate that there was a main effect for valence for both gaze duration and number of fixations (see S4 Fig). Participants gazed longer and more often at AOIs within negative video clips than within positive video clips. In addition, there was a Loneliness by Valence interaction for the number of fixations. As both loneliness and valence only held two levels, we could not use post-hoc comparisons to establish whether the difference in valence held for both groups. Therefore, we ran a Repeated Measures ANOVA with two levels (Valence; positive, negative) for the two loneliness groups separately. Results indicated that participants fixated on the AOIs of the negative video clips more often than the positive video clips in both the lonely (*F*(1,21) = 8.82, *p* = .007, part. η^2^ = .30) and the nonlonely group (*F*(1,21) = 24.74, *p* < .001, part. η^2^ = .54), but this effect was stronger for the nonlonely group. Thus, all participants tended to fixate more often on the facial area of actors in video clips with a negative content compared to video clips with a positive content, and this difference between positive and negative video clips was stronger for nonlonely than for lonely participants. When we did not control for depression and social anxiety, we no longer found a difference between lonely and nonlonely participants.

### Curve Estimations

To examine whether loneliness may only be related to overt visual attention to social cues in the top range of loneliness scores, we used quadratic regression [5]. Within the lonely group, we predicted gazing duration and number of fixations from loneliness scores. None of the quadratic effects were significant, indicating that there was also no relation between loneliness and overt visual attention to social cues for extremely lonely participants.

## Discussion

The goal of the present study was to examine whether lonely individuals show signs of hypervigilance for negative social information. We measured differences between lonely and nonlonely individuals in their overt visual attention to social cues. Results indicated that lonely participants did not differ from non-lonely participants in their overt visual attention to still images. Both lonely and nonlonely participants gazed longer and more often at the eyeregion of the face than at the mouth and nose region (Task 1, Face Task), especially in angry faces. When images with actors expressing happiness, fear, anger, and a neutral expression were presented simultaneously, participants gazed longer and more often at happy images than at other images, and gazed least at the angry images (Task 2, Emotion Array Task). In the third task (Social and Nonsocial Array Task), we found that participants looked longer and more often at social images than at nonsocial images, and looked longer and more often at positive images than at negative images. When participants viewed video clips of positive and negative conversations (Task 4, Video Task), they fixated longer and more often at the facial area of the actors in negative video clips compared to positive video clips. For the number of fixations, this effect was stronger for non-lonely participants than for lonely participants.

These findings seem to imply that lonely individuals do not differ from nonlonely individuals in one of the first steps of processing social cues, namely overt visual attention to social cues. The results in the present study are in contrast with findings in social anxiety research, which provide broad evidence that socially anxious individuals are hypervigilant for social threat. A review article revealed that, with very few exceptions, social anxiety is related to hypervigilance for socially threatening information (specifically facial expressions) in terms of heightened emotion recognition, interpretation, memory and overt and covert attention to threatening information, using a variety of stimuli and paradigms [45]. In social anxiety, evidence is found both for an overall hypervigilance for socially threatening information, and for initial hypervigilance followed by avoidance of these cues [46]. In the two studies that have been conducted on the relation between loneliness and visual attention to socially threatening information, both patterns have been found. Lonely children seemed to show difficulty to disengage from socially threatening information, whereas lonely adults seemed to show initial hypervigilance followed by avoidance of socially threatening cues [5,7]. In the current study, we did not find any link between loneliness and visual attention to social cues, either for positive or negative cues, across a variety of tasks. This could indicate that in loneliness, in contrast with social anxiety, the link with overt attention to social cues does not exist as clearly as in social anxiety.

This could indicate that biased processing of social information in lonely individuals emerges in later stages of social information processing, as seems to be the case in, for instance, depression and borderline personality disorder [25]. For instance, threatening images may be prioritized in encoding irrespective of the visual attention that is given to these cues [47]. In line with this, earlier research showed that lonely individuals seem to have greater activation of the visual cortex in response to viewing social negative images compared to negative images with objects, which indicates that lonely individuals may be more sensitive for these negative social cues [48]. Thus, although lonely individuals did not seem to give more *overt* attention to negative social cues based on the present study, their *covert* attention to these cues may still differ. Processing of these cues might get prioritized over other cues, and memory for these cues may be increased. Regarding our research, lonely participants may have interpreted the cues we presented to them more negatively than the nonlonely participants, may have had biased memory for these cues, or may have showed a different behavioral response to these cues. Indeed, earlier research shows ample evidence for differences such as these in later stages of social information processing, such as a hostile attribution bias, withdrawn behavior, and increased memory for social cues [4,7,49].

Thus, one possible explanation of our findings is that lonely individuals indeed do not show hypervigilance to social cues, but show only biases in other stages of information processing. Alternatively, lonely individuals may in fact be hypervigilant to social cues, but only under specific conditions. First, lonely individuals may only show increased overt attention to specific social cues that convey rejection. Indeed, earlier research indicated that for different forms of psychopathology, different types of stimuli may be relevant [50]. In the few other studies that have looked at the relation between loneliness and overt visual attention to social cues [5,7], the negative cues that were used were videotaped in schoolyards and depicted clear situations of rejection. In contrast, in our study we used a broad body of stimuli that included general facial expressions (Task 1—Face Task and 2—Emotion Array Task), negative social images such as violent behaviors (Task 3—Social and Nonsocial Array Task) and sad or angry conversations (Task 4—Video Task). Hence, these negative cues did not explicitly depict situations of rejection. Possibly, loneliness is only related to hypervigilance towards social threat in terms of rejection.

Second, lonely individuals may only show hypervigilance for negative social cues in situations and towards stimuli in which actual rejection or acceptance is at stake. Earlier research on social anxiety indicated that especially socially threatening information that was self-relevant seemed to activate biased processing of these cues [50]. Indeed, the stimulus material used by Qualter et al. [7] and Bangee et al. [5] showed scenes from a playground. These video clips may have been more ecologically valid than the images and videos used in the present study. Participants may have been more engaged with these video clips, leading to higher identification with the children displayed in these clips. Thus, there may in fact be a difference between lonely and nonlonely individuals in their overt visual attention to social cues, but we were unable to detect it as our tasks may not have been socially relevant enough to trigger hypervigilance for negative social cues. Some researchers argue that the study of social gaze behavior could benefit from designs using active participation in social interaction, as processing of more life-like social information could differ from information as being processed in a lab environment [51]. Future research is obviously needed to examine whether or not loneliness is in fact related to differential overt visual attention to social cues in real life interactions.

Third, loneliness may be only related to hypervigilance to negative social information for certain lonely individuals. For instance, it might be possible that loneliness is only related to hypervigilance for negative social cues in certain age groups whose social and emotional skills are still developing. Earlier research showed differences in patterns of overt visual attention to rejection cues between lonely children and older adolescents. Biases in overt visual attention to social cues may only arise in age-groups that were not included in the present study, such as children or older adolescents [5,7]. In addition, hypervigilance to negative social information may only arise in certain types of lonely individuals. For instance, possibly everyone who experiences some threat to the belonging regulation system may show increased attention to social cues in general, but this may shift to hypervigilance for negative social cues only for chronically lonely individuals [3,4,16,52]. In the present study, we only included young adults, and due to the correlational nature of our data, we were unable to distinguish temporary lonely from chronically lonely individuals. Future research could explore the possibility that only certain types of lonely individuals may be hypervigilant for negative social cues.

One of the strong points of the present study is the use of four different eye-tracking tasks, which allowed us to do a within-sample replication of our finding that loneliness does not seem to be related to overt visual attention to social cues. The overall main effects (i.e., gazing behavior of the entire sample) were in the direction we expect for a healthy population. For instance, in accordance with earlier research, we found highest gazing towards the eye region of the face in Task 1 (Face Task), and preference for social images over nonsocial images in Task 3 (Social and Nonsocial Task) [53,54]. Thus, we think that our tasks were well designed and suitable to detect differences in overt attention to negative social cues. Of course, because we only used one sample for all four tasks, there still is a possibility that our findings are specific for our sample, although we have no reason to assume that our sample of lonely individuals was not comparable to other samples.

One of the limitations of the present study is that we used only highly educated female participants. Although we have no reason to assume that visual attention to social cues may be different for males, or individuals from other educational backgrounds, the sample in the present study may not be generalizable to the general population. Therefore, future research should explore the relation between loneliness and visual attention in different samples such as males or individuals from a lower educational background. Another limitation of the present study is the use of non-lonely participants as a contrast group. Research on loneliness thus far has been aimed at the most lonely people. As a result, we cannot draw conclusions about possible differences between people who experience average, high or low feelings of loneliness. Future research might address the issue of whether nonlonely participants may show certain biases as well.

Future research should explore the possibility that differences in processing of social information may occur in later stages of social information processing. Moreover, in a real-life interaction task, lonely and nonlonely participants could be exposed to a confederate displaying negative or positive social cues. In such an experiment, behavioral responses of lonely and nonlonely participants could be measured. Future research could also benefit from combining an eye-tracking approach with an EEG approach. By combining these approaches, future research could reveal differences in overt and covert processing of social information by lonely and nonlonely individuals. Additionally, future research could examine whether loneliness might be related to hypervigilance to negative social cues, but only under specific conditions.

### Conclusion

All in all, we did not find evidence that lonely individuals are hypervigilant for negative social cues. Both lonely and nonlonely individuals tend to gaze longer and more often at areas that convey most social information, such as the facial area and specifically the eye region. This could indicate that biased processing of social information in lonely individuals emerges in a later stage of social information processing. Alternatively, hypervigilance for negative social cues may only become apparent for specific cues (e.g., rejection cues), in specific situations (e.g., situations in which actual rejection or acceptance is at stake), or for certain lonely individuals (e.g., chronically lonely individuals). Notwithstanding alternative explanations, based on our research, we have no evidence for a relation between loneliness and hypervigilance for negative social cues.

## Supporting Information

S1 Table Slide Numbers for IAPS Images Task 3. (DOCX)

S2 Table Mixed Model ANOVA Results for Gaze Duration in ms and Number of Fixations for All Tasks Including Covariates and Errors. (DOCX)

S3 Table Means and Standard Deviations for Percentage of First Fixations for Total Sample, Lonely and Nonlonely Participants for Task 2 (Emotional Array Task) and Task 3. (DOCX)

S1 Fig Corrected Total Fixation Duration at Eyes, Nose and Mouth in Task 1 (Face Task) for Lonely and Nonlonely Participants. (TIF)

S2 Fig Total Fixation Duration at Emotions in Task 2 (Emotion Array Task) for Lonely and Nonlonely Participants. (TIF)

S3 Fig Total Fixation Duration at Different Images in Task 3 (Social and Nonsocial Array Task) for Lonely and Nonlonely Participants. (TIF)

S4 Fig Total Fixation Duration at AOIs in Task 4 (Video Task) for Lonely and Nonlonely Participants. (TIF)
