# Supplementary material for: Loneliness and Hypervigilance to Social Cues in Females: An Eye-Tracking Study
Source: PLoS One. 2015 Apr 27;10(4):e0125141. doi: 10.1371/journal.pone.0125141 (PMC4410954; doi:10.1371/journal.pone.0125141)
Supplement: S1 Table — (DOCX) [file pone.0125141.s001.docx]

Table S3. Frame Numbers for IAPS Images Task 3.

| Positive | |  | Negative | |
| --- | --- | --- | --- | --- |
| Social | Nonsocial |  | Social | Nonsocial |
| 1340 | 1410 |  | 2278 | 1114 |
| 2151 | 1440 |  | 2312 | 1202 |
| 2156 | 1540 |  | 2455 | 1270 |
| 2158 | 1590 |  | 2456 | 1275 |
| 2222 | 1722 |  | 2590 | 6020 |
| 2274 | 5210 |  | 2703 | 6610 |
| 2340 | 5665 |  | 2718 | 6800 |
| 2341 | 5814 |  | 2900 | 7078 |
| 2347 | 5825 |  | 6242 | 7136 |
| 2373 | 5990 |  | 6571 | 7520 |
| 2398 | 7200 |  | 6832 | 7521 |
| 4628 | 7250 |  | 9220 | 9000 |
| 4640 | 7260 |  | 9415 | 9001 |
| 5831 | 7330 |  | 9419 | 9610 |
| 7499 | 7390 |  | 9424 | 9623 |
| 8032 | 7405 |  | 9520 | 9830 |
| 8040 | 7430 |  | 9530 | 9832 |
| 8380 | 7492 |  | 9900 | 9909 |
| 8400 | 8500 |  | 9926 | 9912 |
| 8420 | 8501 |  | 9927 | 9930 |
