# Supplementary material for: Loneliness and Hypervigilance to Social Cues in Females: An Eye-Tracking Study
Source: PLoS One. 2015 Apr 27;10(4):e0125141. doi: 10.1371/journal.pone.0125141 (PMC4410954; doi:10.1371/journal.pone.0125141)
Supplement: S2 Table — (DOCX) [file pone.0125141.s002.docx]

Table S2. Mixed Model ANOVA Results for Gaze Duration in ms and Number of Fixations for All Tasks Including Covariates and Errors.

|  | Fixation Duration | | | |  | N Fixations | | | |
| --- | --- | --- | --- | --- | --- | --- | --- | --- | --- |
|  | *df* | *MS* | *F* | η2 |  | *df* | *MS* | *F* | η2 |
| Task1 |  |  |  |  |  |  |  |  |  |
| Social Anxiety | 1.00 | 4.68 | .40 | .01 |  | 1.00 | 5.69 | .52 | .01 |
| Depression | 1.00 | .45 | .04 | .00 |  | 1.00 | 1.22 | .11 | .00 |
| Loneliness | 1.00 | .20 | .02 | .00 |  | 1.00 | 2.43 | .22 | .00 |
| Error(between) | 46.00 | 11.63 |  |  |  | 46.00 | 1.94 |  |  |
| AOI | 1.78 | 884.60 | 38.49*** | .46 |  | 1.67 | 7558.60 | 33.92*** | .42 |
| AOI x Soc Anxiety | 1.78 | 48.32 | .21 | .00 |  | 1.67 | 41.62 | .19 | .00 |
| AOI x Depression | 1.78 | 239.22 | 1.04 | .02 |  | 1.67 | 145.19 | .65 | .01 |
| AOI x Loneliness | 1.78 | 6.31 | .03 | .00 |  | 1.67 | 4.40 | .18 | .00 |
| Error(AOI) | 81.80 | 229.69 |  |  |  | 76.82 | 222.81 |  |  |
| Emotion | 4.00 | 74.32 | 16.59*** | .27 |  | 4.00 | 68.35 | 2.37*** | .31 |
| Emotion x Soc Anxiety | 4.00 | 1.27 | .28 | .01 |  | 4.00 | 1.29 | .38 | .01 |
| Emotion x Depresion | 4.00 | 1.02 | .23 | .00 |  | 4.00 | .43 | .13 | .00 |
| Emotion x Loneliness | 4.00 | 2.99 | .67 | .01 |  | 4.00 | 3.91 | 1.16 | .02 |
| Error(Emotion) | 184.00 | 4.48 |  |  |  | 184.00 | 3.36 |  |  |
| AOI x Emotion | 7.55 | 25.95 | 2.64** | .05 |  | 7.63 | 16.57 | 2.27* | .05 |
| AOI x Emotion x Soc Anxiety | 7.55 | 1.30 | 1.05 | .02 |  | 7.63 | 6.02 | .83 | .02 |
| AOI x Emotion x Depression | 7.55 | 4.30 | .44 | .01 |  | 7.63 | 3.75 | .51 | .01 |
| AOI x Emotion x Loneliness | 7.55 | 14.72 | 1.50 | .03 |  | 7.63 | 9.34 | 1.28 | .03 |
| Error(AOI*Emotion) | 347.21 | 9.85 |  |  |  | 351.08 | 7.29 |  |  |
| Taak 2 |  |  |  |  |  |  |  |  |  |
| Social Anxiety | 1.00 | 21643.42 | .72 | .02 |  | 1.00 | .00 | .00 | .00 |
| Depression | 1.00 | 50994.43 | 1.70 | .04 |  | 1.00 | .19 | .37 | .01 |
| Loneliness | 1.00 | 8971.87 | .30 | .01 |  | 1.00 | .74 | 1.46 | .03 |
| Error(between) | 46.00 | 29979.88 |  |  |  | 46.00 | .51 |  |  |
| Emotion | 2.82 | 1208253.08 | 16.42*** | .26 |  | 2.95 | 9.69 | 18.06*** | .28 |
| Emotion x Soc Anxiety | 2.82 | 11379.47 | 1.55 | .03 |  | 2.95 | 1.03 | 1.93 | .04 |
| Emotion x Depression | 2.82 | 112149.23 | 1.52 | .03 |  | 2.95 | .89 | 1.65 | .03 |
| Emotion x Loneliness | 2.82 | 43094.94 | .59 | .01 |  | 2.95 | .16 | .30 | .01 |
| Error(Emotion) | 129.90 | 73582.83 |  |  |  | 135.60 | .54 |  |  |
| Taak 3 |  |  |  |  |  |  |  |  |  |
| Social Anxiety | 1.00 | 5717.22 | .11 | .00 |  | 1.00 | .00 | .01 | .00 |
| Depression | 1.00 | 13497.22 | .27 | .01 |  | 1.00 | .14 | .20 | .00 |
| Loneliness | 1.00 | 19356.33 | .39 | .01 |  | 1.00 | .10 | .14 | .00 |
| Error (between) | 46.00 | 50012.21 |  |  |  | 46.00 | .69 |  |  |
| Image | 2.77 | 1922352.40 | 87.71*** | .66 |  | 2.75 | 261.12 | 109.88*** | .70 |
| Image x Social Anxiety | 2.77 | 508915.12 | 2.32 | .05 |  | 2.75 | 4.87 | 2.05 | .04 |
| Image x Depression | 2.77 | 88083.00 | .40 | .01 |  | 2.75 | .26 | .11 | .00 |
| Image x Loneliness | 2.77 | 199939.75 | .91 | .02 |  | 2.75 | 2.62 | 1.10 | .02 |
| Error (Image) | 127.39 | 219169.91 |  |  |  | 126.33 | 2.38 |  |  |
| Taak 4 |  |  |  |  |  |  |  |  |  |
| Social Anxiety | 1.00 | 67818.04 | .00 | .00 |  | 1.00 | 1.81 | .01 | .00 |
| Depression | 1.00 | 16879091.38 | .43 | .01 |  | 1.00 | 2.52 | .08 | .00 |
| Loneliness | 1.00 | 9371122.41 | .24 | .01 |  | 1.00 | 621.65 | 2.56 | .05 |
| Error (between) | 44.00 | 39436735.48 |  |  |  | 44.00 | 243.10 |  |  |
| Valence | 1.00 | 98067836.94 | 86.87*** | .66 |  | 1.00 | 441.96 | 7.29*** | .62 |
| Valence x Soc Anxiety | 1.00 | 3535502.68 | 3.13 | .07 |  | 1.00 | 32.58 | 5.18* | .11 |
| Valence x Depression | 1.00 | 368618.89 | .33 | .01 |  | 1.00 | 11.56 | 1.84 | .04 |
| Valence x Loneliness | 1.00 | 1613139.35 | 1.43 | .03 |  | 1.00 | 4.50 | 6.44* | .13 |
| Error (Valence) | 44.00 | 1128903.45 |  |  |  | 44.00 | 6.29 |  |  |

* *p* < .05. ** *p* < .01. *** *p* < .001.
