# Supplementary material for: Loneliness and Hypervigilance to Social Cues in Females: An Eye-Tracking Study
Source: PLoS One. 2015 Apr 27;10(4):e0125141. doi: 10.1371/journal.pone.0125141 (PMC4410954; doi:10.1371/journal.pone.0125141)
Supplement: S3 Table — (DOCX) [file pone.0125141.s003.docx]

**Table S1.** Means and Standard Deviations for Percentage of First Fixations for Total Sample, Lonely and Nonlonely Participants for Task 2 and Task 3.

|  | Total | |  | Lonely | |  | Nonlonely | |
| --- | --- | --- | --- | --- | --- | --- | --- | --- |
|  | *M* | *SD* |  | *M* | *SD* |  | *M* | *SD* |
| Task 2 |  |  |  |  |  |  |  |  |
| Anger | 25.48 | 7.41 |  | 26.18 | 7.10 |  | 24.78 | 7.79 |
| Fear | 27.66 | 9.13 |  | 26.73 | 8.43 |  | 28.60 | 9.86 |
| Happiness | 20.74 | 8.41 |  | 21.45 | 9.14 |  | 20.03 | 7.74 |
| Neutral | 26.12 | 7.40 |  | 25.64 | 7.75 |  | 26.60 | 7.16 |
| Task 3 |  |  |  |  |  |  |  |  |
| Pos Soc | 37.20 | 9.70 |  | 37.00 | 9.13 |  | 37.40 | 10.42 |
| Neg Soc | 30.80 | 10.61 |  | 31.00 | 10.70 |  | 30.60 | 10.74 |
| Pos NonSoc | 18.30 | 9.29 |  | 18.60 | 7.29 |  | 18.00 | 11.09 |
| Neg NonSoc | 13.70 | 7.61 |  | 13.40 | 6.24 |  | 14.00 | 8.90 |

*Note.*  *N* = 25 for lonely and nonlonely group for both tasks.
