# Supplementary figures and images for: Loneliness and Hypervigilance to Social Cues in Females: An Eye-Tracking Study
Source: PLoS One. 2015 Apr 27;10(4):e0125141. doi: 10.1371/journal.pone.0125141 (PMC4410954; doi:10.1371/journal.pone.0125141)

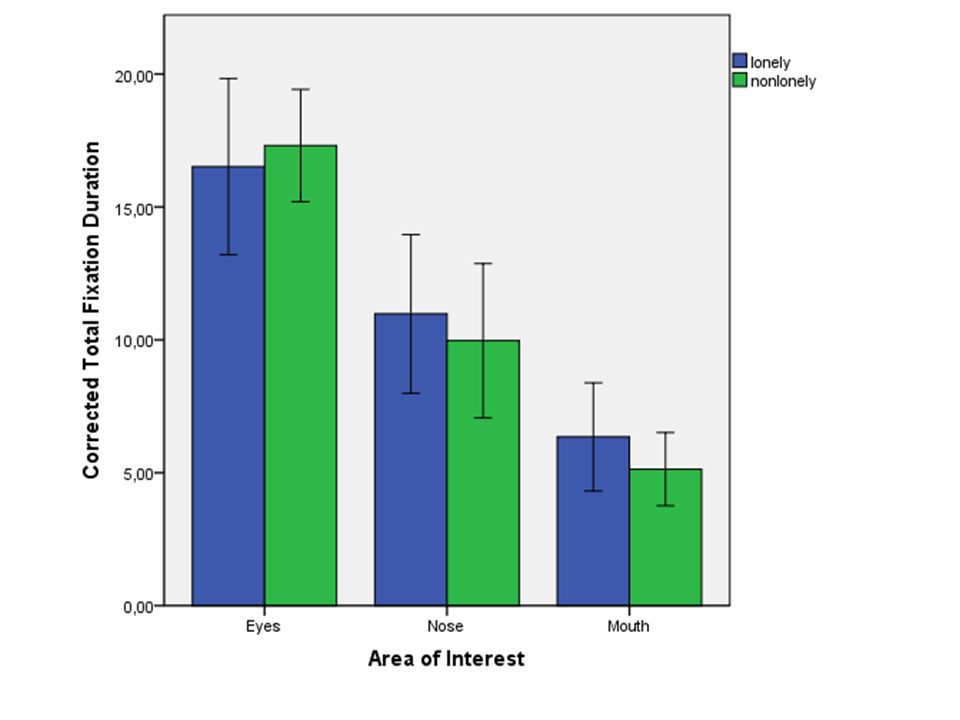

Supplement: S1 Fig — (TIF) [file pone.0125141.s004.tif]

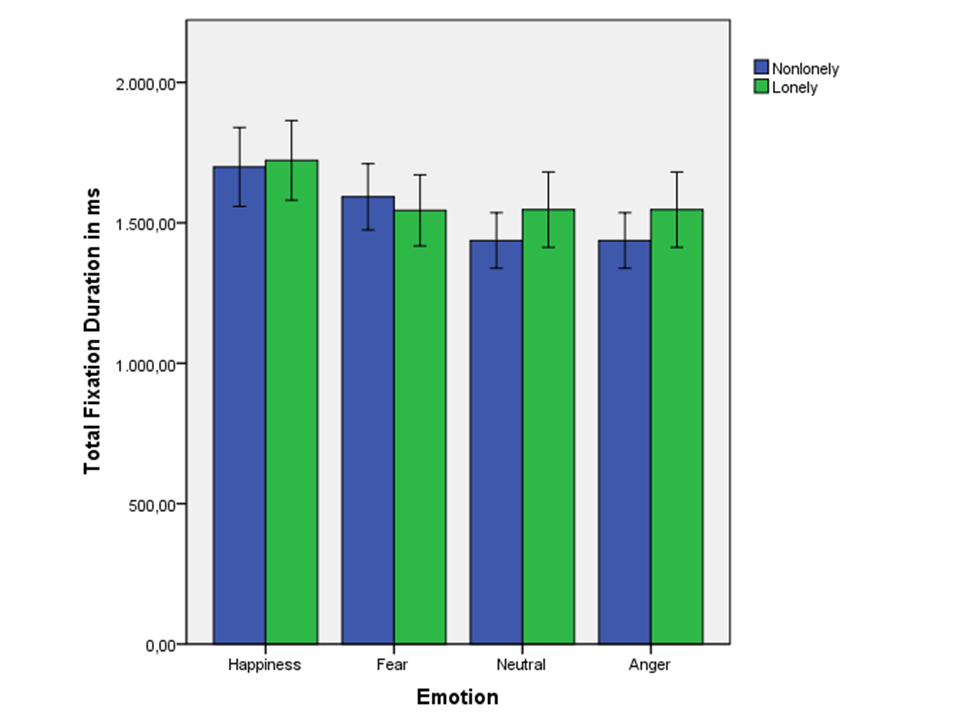

Supplement: S2 Fig — (TIF) [file pone.0125141.s005.tif]

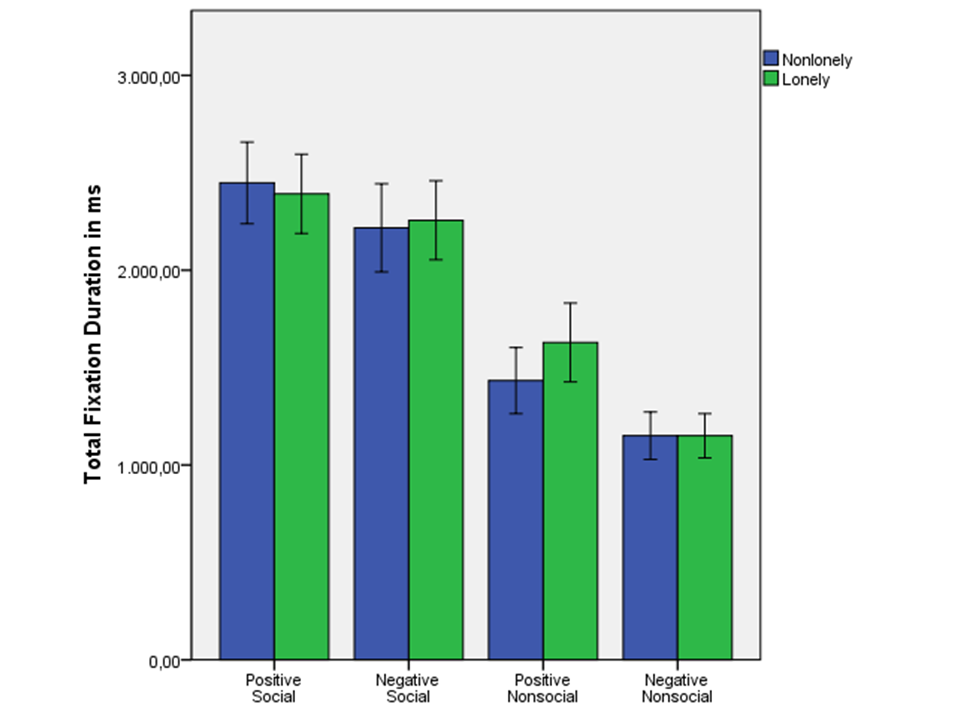

Supplement: S3 Fig — (TIF) [file pone.0125141.s006.tif]

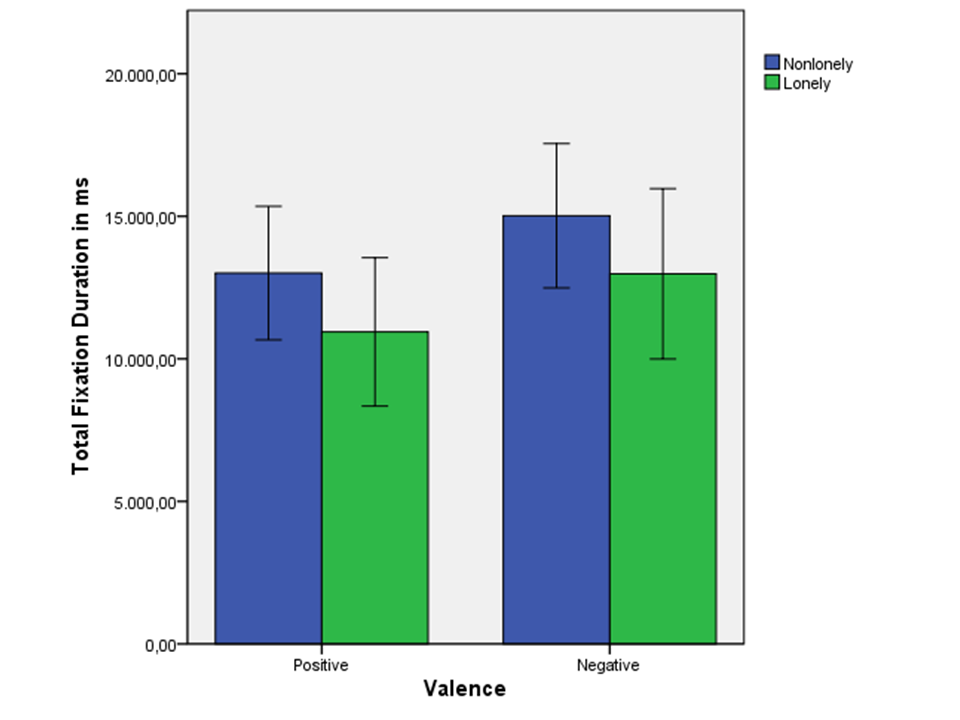

Supplement: S4 Fig — (TIF) [file pone.0125141.s007.tif]
